# Dynamics of Suitable Habitats for Typical Predators and Prey on the Qinghai‐Tibet Plateau Driven by Climate Change: A Case Study of Tibetan Fox, Red Fox, and Plateau Pika

**DOI:** 10.1002/ece3.71295

**Published:** 2025-04-18

**Authors:** Jingjie Zhang, Feng Jiang, Hongmei Gao, Haifeng Gu, Pengfei Song, Tongzuo Zhang

**Affiliations:** ^1^ State Key Laboratory of Plateau Ecology and Agriculture Qinghai University Xining Qinghai China; ^2^ Key Laboratory of Adaptation and Evolution of Plateau Biota, Northwest Institute of Plateau Biology Chinese Academy of Sciences Xining China; ^3^ Qinghai Provincial Key Laboratory of Animal Ecological Genomics, Northwest Institute of Plateau Biology Chinese Academy of Sciences Xining China

**Keywords:** centroid shift, climate change, predators, prey, Qinghai‐Tibet plateau

## Abstract

The Qinghai‐Tibet Plateau (QTP) is a biodiversity hotspot highly sensitive to global climate change. The Tibetan fox ( *Vulpes ferrilata* ), red fox ( *V. vulpes* ), and plateau pika ( *Ochotona curzoniae* ) are key species of the plateau, serving as typical representatives of predators and prey among its diverse wildlife. To evaluate the impact of climate change, we employed the maximum entropy model with 1237 distribution points and various environmental variables to predict habitat suitability under three global climate models and four representative concentration pathways for the 2050s and 2070s. The results revealed that the suitable habitats for two predators were projected to decline, with reductions ranging from 0.23% to 5.64% and 4.12% to 6.63%, respectively, with most reductions occurring in the central‐western and southern regions of the QTP. The decline was anticipated to be more pronounced in the 2070s compared to the 2050s. Conversely, the suitable habitat for prey species, plateau pikas, was expected to experience only a slight decrease (0.45%–0.98%) under scenarios of moderate greenhouse gas emissions. Habitat centroid analyses indicated a consistent northward migration of suitable areas for both predators and prey in response to climate change on the QTP. Furthermore, future overlap analysis between predator and prey habitats showed uncertain trends; however, the overlap between the Tibetan fox and Plateau pika habitats was notably lower compared to that of the red fox and plateau pika habitats. Regarding the current conservation efforts of both predators and prey, evaluation results highlighted the critical significant role of Sanjiangyuan National Park, China's first national park located in Qinghai Province, and Qiangtang Nature Reserve in Xizang as critical areas for the protection of these species on the QTP in China. The findings and methodologies of this research hold significant reference value for the conservation of predator and prey habitats in other global biodiversity hotspots.

## 1 Introduction

Climate change has emerged as a primary driver of global ecosystem transformations. According to the *Fifth Assessment Report of the IPCC* (Adopted 2014), the global average surface temperature increased by approximately 0.85°C between 1880 and 2012 due to persistent greenhouse gas emissions. By the end of this century, the average temperature is projected to rise further by 1.0°C–3.7°C. Warming temperatures and frequent extreme weather events have directly impacted species distributions, habitat suitability, and migration patterns. Many organisms, particularly temperature‐sensitive species, are gradually migrating toward higher latitudes and elevations in search of suitable habitats (Chen et al. 2011; Root et al. 2003). This trend has triggered ecological ripple effects on a global scale, altering interspecies interactions and even altering biodiversity patterns within habitats (Parmesan and Yohe 2003). In addition, as the pace of climate change exceeds the speed of species' evolutionary and adaptive responses, many species face intensified competition and survival pressures in rapidly changing environments (Bellard et al. 2012).

One region particularly vulnerable to these changes is the Qinghai‐Tibet Plateau (QTP), which has seen profound and complex ecological shifts as a result of global warming. The climate and ecosystem of the QTP are unique, and it is an important habitat for many species. In recent decades, the temperature of the QTP has risen significantly, resulting in the continuous upward movement of the snow line and the significant degradation of permafrost, with serious ecological consequences (Qiu 2008). Plateau warming has led to an increase in drought frequency and unstable precipitation patterns, which have changed the distribution pattern of plants and ecosystem structure, and led to the survival pressure of native species adapted to alpine conditions. Furthermore, habitat fragmentation and intensified niche competition compel some species to inhabit less optimal areas, threatening overall ecosystem stability (Loarie et al. 2009).

The Tibetan fox ( *Vulpes ferrilata* ), red fox ( *Vulpes vulpes* ) and Plateau pika ( *Ochotona curzoniae* ) are keystone species in the QTP ecosystem, forming a characteristic predator–prey relationship. The plateau pika, a small lagomorph, is widely distributed in the alpine grasslands of the QTP. Studies have shown that varying intensities of plateau pika disturbance exert differential effects on the functioning of grassland ecosystems on the QTP. Low to moderate disturbance levels increase aboveground biomass and soil organic carbon, whereas disturbances beyond moderate levels lead to declines in species richness and aboveground biomass (Cui et al. 2024). Data indicated that the burrowing activity of plateau pikas reduces topsoil moisture and hardness, thereby increasing soil erosion, hindering vegetation recovery (Chen et al. 2017), and enhancing ecosystem carbon emissions (Qin et al. 2015). Other studies suggest that the presence of plateau pikas stimulates microbial activity, improves the decomposition of organic matter and soil nitrogen availability (Villarreal et al. 2008; Zhang et al. 2016), and increases the organic matter content in the topsoil (Li and Zhang 2006). Consequently, the ecological role of this species on the QTP remains controversial. Plateau pikas, as vital primary consumers, are the main food source for the Tibetan and red foxes (Harris et al. 2014). In particular, plateau pika constitutes a high proportion of the Tibetan fox's diet (Hacker et al. 2022; Lu et al. 2023; Smith et al. 2019). The Tibetan fox is highly adapted to alpine meadows and grasslands (Clark et al. 2008), whereas the red fox exhibits a broader ecological niche, primarily reflected in its dietary diversity (Hacker et al. 2022). The two species coexist in some areas of the plateau and may have a certain resource competition (Statham et al. 2014). Collectively, these three species reveal key ecological processes on the Qinghai‐Tibet Plateau—such as predator–prey interactions, ecological disturbances, and species adaptability—thus demonstrating significant representativeness and research value for this study.

This study will employ the Maximum Entropy (Maxent) model to simulate species distributions. Based on the principle of maximum entropy, the model establishes a probability distribution using species occurrence data and environmental variables, thereby enabling accurate predictions of potential species distributions. Its advantages include low sample size requirements, ease of operation, and excellent predictive performance, which have led to its widespread application in ecological niche modeling, species distribution forecasting, and climate change impact assessments (Elith et al. 2011; Phillips et al. 2006). Numerous studies have demonstrated that the Maxent model consistently yields highly accurate predictions across various regions and species, effectively supporting biodiversity conservation and ecological management decisions (Merow et al. 2013).

Due to the increasingly significant impact of climate change on alpine ecosystems, studying the reliance of these foxes on plateau pikas provides a model for understanding how climate stressors influence food web dynamics and niche distribution. By analyzing the impact of climate change on the food chain, we can better understand how the distribution and niches of the three species will change under climate pressure in the future. This will help us reveal how climate change affects species interactions and the stability of regional ecosystems. These insights are crucial for informing biodiversity conservation strategies on the QTP.

## 2 Materials and Methods

### 2.1 Study Area

The QTP (25° N‐40° N, 74° E‐104° E) known as the “Roof of the World,” has a distinctive climate characterized by high altitude, cold temperatures, and low oxygen levels. Its ecosystems, ranging from alpine meadows to cold deserts, support significant biodiversity. This plateau extends westward from the Pamir Plateau, eastward to the Hengduan Mountains, southward to the southern edge of the Himalayas, and northward to the northern reaches of the Kunlun and Qilian Mountains (Liu et al. 2022).

### 2.2 Species Occurrence Data and Processing

**FIGURE 1:**
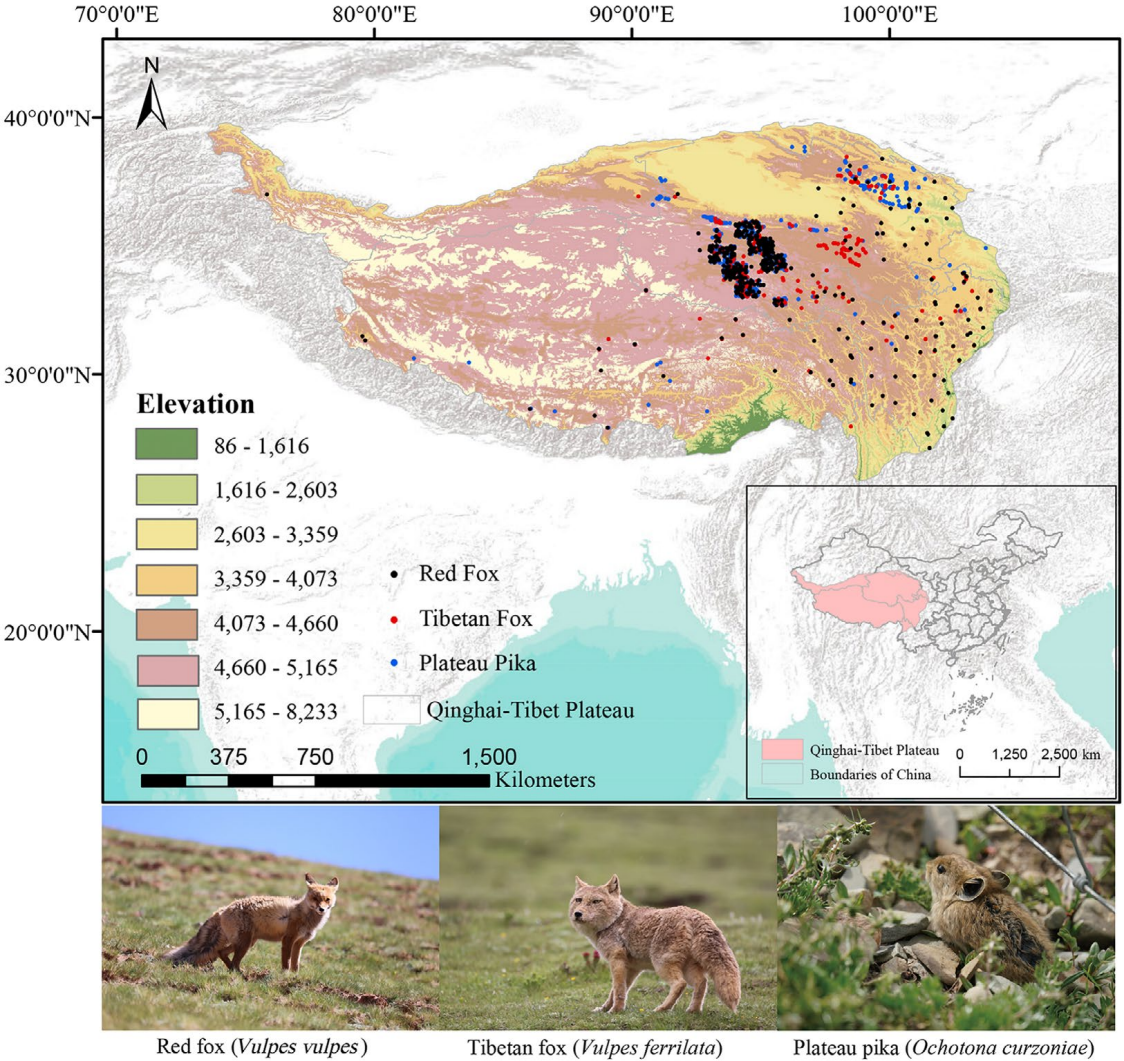
Occurrence points and pictures of Tibetan fox, red fox, and plateau pika on the Qinghai‐Tibet Plateau (the photographer is Tongzuo Zhang).

Species occurrence data for the predators, Tibetan fox and red fox, and their prey, Plateau pika, were primarily collected from large‐scale terrestrial wildlife surveys conducted from 2015 to 2024 using methods such as sampling points, transects, and quadrats. Additional data were obtained from the Global Biodiversity Information Facility (GBIF) database (https://www.gbif.org) and the Species Diversity Data Platform (SDDP) database (especies.cn). Since the SDDP data did not provide detailed latitude and longitude coordinates for occurrence points, the geographic locations were determined through map‐based positioning. Additionally, to mitigate the potential negative effects of spatial autocorrelation among occurrence data, only a single record was retained within each high‐resolution (30 × 30 arc‐seconds) grid cell. Initially, there were 449, 390, and 622 occurrence points for the Red fox, Tibetan fox, and Plateau pika, respectively. After data processing, 442, 258, and 537 high‐precision occurrence points were retained for subsequent analysis (Figure 1).

### 2.3 Environmental Variables and Selection

The present study utilized 19 bioclimatic variables (Table S1) from the WorldClim Global Climate Data platform (http://www.worldclim.org/), based on the average values from 1970 to 2000, with a spatial resolution of 30 arc‐seconds (approximately 1 km). For simulating species distribution under future climate conditions, 19 bioclimatic variables for the years 2050s (average for 2041–2060) and 2070s (average for 2061–2080) were also obtained from WorldClim. Given the inherent uncertainties in species distribution models, employing multiple future climate scenarios provides a more robust prediction of potential shifts in species distribution due to climate change (Thuiller et al. 2005). We applied three distinct global climate models CCSM4, HadGEM2‐ES, and MIROC5 to assess distributional shifts for three species under changing climate conditions. To enhance prediction accuracy and breadth, this study employed four representative concentration pathway (RCP) scenarios, including a stringent mitigation scenario (RCP2.6), two intermediate scenarios (RCP4.5 and RCP6.0), and a high greenhouse gas emissions scenario (RCP8.5). In the process of selecting climate variables, we excluded one variable from each pair with a high Pearson correlation coefficient (|r| ≥ 0.8), thereby minimizing the potential for multicollinearity issues (Gebrewahid et al. 2020; Harrington et al. 2018). More generalizable environmental variables were retained and subsequently integrated into the model for further computational analysis. Through variable selection, seven bioclimatic factors were included in the models for both Tibetan fox and red fox, while eight factors were included for Plateau pika (Figure S1).

### 2.4 Species Distribution Model Building

In this study, we employed the MaxEnt model, known for its high predictive performance and stability, to forecast the current and future habitat distributions of the three species under investigation. Studies suggest that optimizing parameters enhances the quality of MaxEnt outputs. To achieve this, we used ENMTools 1.4.3 to calculate corrected Akaike Information Criterion (AICc) values for various combinations of regularization multipliers (RM) and feature classes (FC). Parameters with lower AICc values (Muscarella et al. 2014) and smooth response curves were selected. The optimal parameter combinations for the models of Tibetan fox, red fox, and Plateau pika are presented in Table S1. For model training, 75% of occurrence points were used for training, and 25% for testing (Tanner et al. 2017), with 10 replicates. Model performance was assessed using the area under the curve (AUC) of the receiver operating characteristic curve, where higher AUC values indicate better predictive accuracy (Gonzalez et al. 2011; Jiang et al. 2020). The average test AUC values for replicate runs almost exceeded 0.8 (Figures S2–S4), indicating good model performance (Zhang et al. 2024). Species distribution layers were classified into suitable or unsuitable habitats based on the mean logistic threshold of Maximum Training Sensitivity Plus Specificity (MTSPS) (Ma et al. 2022; Qiu et al. 2023). Grids with probability values exceeding the threshold were considered suitable habitats.

### 2.5 Suitable Habitat Changes and Transfer Analysis

We used ArcGIS software to overlay current and future species distribution layers to identify stable, expanding, and degraded areas under climate change in this study. Additionally, we calculated the centroids of species distributions under current and future climate scenarios using the following formula: $\bar{X\;}$ = $\sum_{i=1}^{n}M_{i}X_{i}/\sum_{i=1}^{n}M_{i}$ and $\bar{Y}$ = $\sum_{i=1}^{n}M_{i}Y_{i}/\sum_{i=1}^{n}M_{i}$ (He et al. 2011; Jiang et al. 2020).

We assumed that the species' suitable distribution area is composed of *i* regions, where *M*_*i*_ represents the mass of each region. *Mi* was calculated as *M*_*i*_ *= ρ*_i *_ *Si*, where *ρ*_*i*_ was the probability of species occurrence; it can be obtained from habitat suitability values, and *S*_*i*_ was the area of region *i*. *X*_*i*_ and *Y*_*i*_ denote the latitude and longitude of region *i*, respectively.

### 2.6 Changes in Overlap Areas Between Predators and Prey and Analysis of Conservation Gaps

In this study, we utilized ArcGIS software to overlay current and future distribution maps of two predator–prey pairs (Tibetan fox and red fox as predators, plateau pika as prey) and two predators to assess changes in the proportion of suitable habitat overlap. Additionally, we analyzed the current distribution of the three species within national nature reserves on the QTP to evaluate the effectiveness of existing conservation efforts and identify gaps in protection. This approach provides insights into habitat dynamics and informs strategic conservation planning.

## 3 Results

### 3.1 Analysis of Key Environmental Variables Affecting the Distribution of Three Species and Their Variability

**FIGURE 2:**
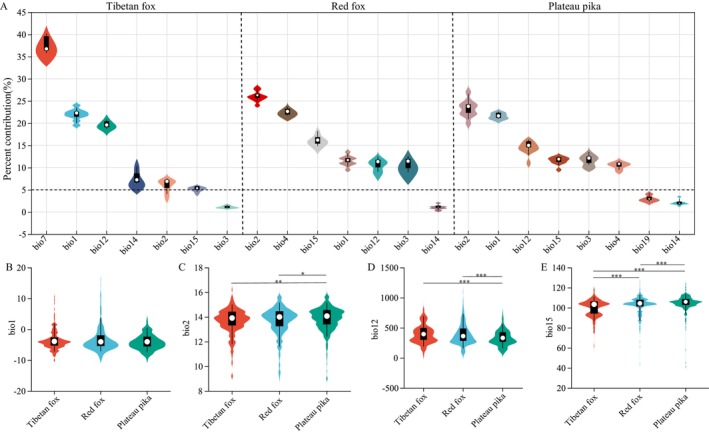
Contribution rates of climate factors for the three species (A) and comparative analysis of annual mean temperature (B), mean diurnal temperature range (C), annual precipitation (D), and precipitation seasonality (E) among the three species.

Contribution rates (Table S2) identified annual mean temperature (bio1) and annual precipitation (bio12) as major influences in three species, alongside temperature seasonality (bio4), temperature annual range (bio7), and mean diurnal temperature range (bio2) for specific species. Results showed that temperature had a stronger influence than precipitation for all three species (Figure 2). Comparing key bioclimatic factors with high contributions revealed that, apart from annual mean temperature (bio1), significant differences were observed in mean diurnal temperature range (bio2), annual precipitation (bio12), and precipitation seasonality (bio15) between predators and prey. The plateau pika demonstrates a significantly higher adaptation to the mean diurnal temperature range compared to both the red fox and Tibetan fox. Additionally, the plateau pika's suitable habitat exhibits greater precipitation seasonality variability than the other two species. In contrast, the Tibetan and red foxes' suitable habitats experience significantly higher annual precipitation than that of the plateau pika. These differences underscore the species' distinct ecological requirements, reflecting their adaptation to varying climatic factors across the Tibetan Plateau.

### 3.2 Changes in Species Distribution Caused by Climate Change

**FIGURE 3:**
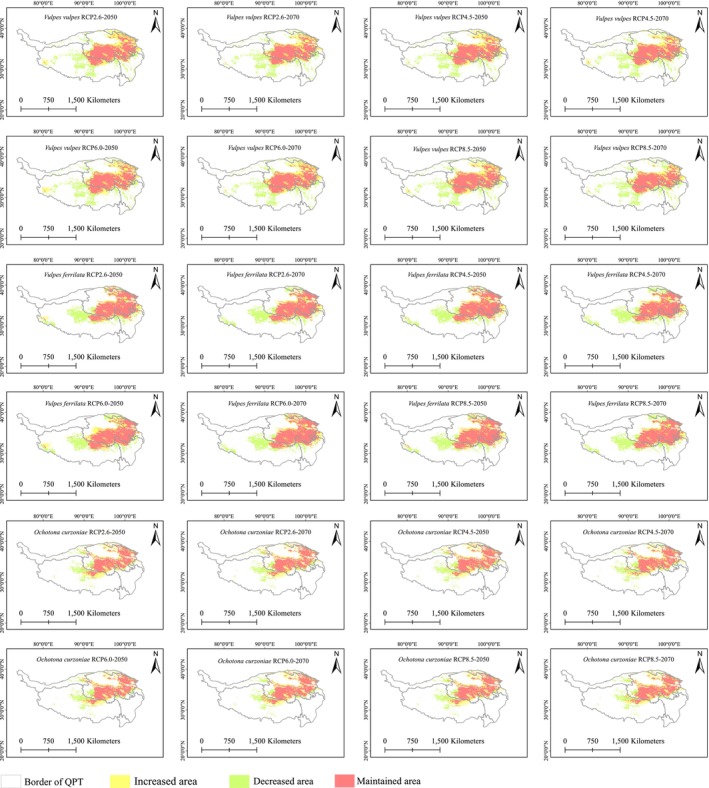
Changes in suitable distribution areas for the three species under different years and climate scenarios.

Both red fox and Tibetan fox suitable habitats were projected to decrease under future climate change scenarios, with area reductions ranging from 0.23% to 5.64% and 4.12% to 6.63%, respectively. The prediction results showed that the suitable habitat area for species in the 2070s is generally smaller than in the 2050s. The plateau pika's future habitat experienced a slight reduction (0.45%–0.98%) under scenarios RCP2.6 (2070s), RCP4.5 (2050s and 2070s), and RCP6.0 (2050s), but its habitat also expanded by 0.04%–0.66% in other climate scenarios (Table 1). Notably, the highest expansion rate occurs in the 2050s under the strict greenhouse gas mitigation scenario (RCP2.6). Habitat change results indicated a loss of habitats in the western part of the Tibetan Plateau, particularly in the central region (Figure 3).

**TABLE 1:** Proportion of suitable habitat area increase or decrease for the three species under future climate change.

Proportion/%	RCP2.6–2050s	RCP2.6–2070s	RCP4.5–2050s	RCP4.5–2070s	RCP6.0–2050s	RCP6.0–2070s	RCP8.5–2050s	RCP8.5–2070s
Red fox	−0.23	−3.98	−2.89	−3.76	−2.46	−2.41	−2.18	−5.64
Tibetan fox	−4.12	−6.13	−4.83	−6.35	−4.30	−4.18	−4.63	−6.63
Plateau pika	0.66	−0. 98	−0.45	−0.63	−0.52	0.25	0.19	0.04

### 3.3 Shift of the Distribution Centroid Based on Future Climatic Conditions

**FIGURE 4:**
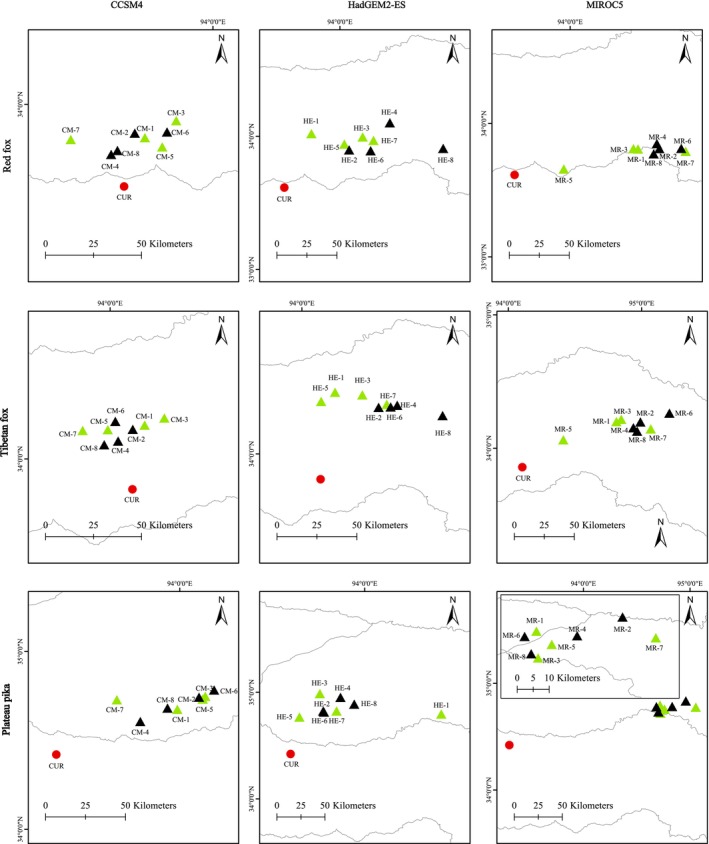
Shifts in the centroids of suitable distribution areas for the three species under climate change.

By calculating the centroid coordinates of the current and future suitable habitats for the three species under the influence of climate change, we found that the centroids shifted toward higher latitudes and exhibited a northeastward trend under all scenarios. The results indicated that the future latitudinal ranges of the centroids for the red fox, Tibetan fox, and plateau pika were 33.66° N–34.10° N, 34.61° N–34.99° N, and 34.06° N–34.35° N, respectively, with northward migration spanning 0.04°–0.57°. Based on straight‐line distances, the migration distances for the red fox, Tibetan fox, and plateau pika ranged from 17.65 to 120.34 km, 25.89 to 111.17 km, and 52.33 to 164.85 km, respectively (Figure 4).

### 3.4 Variations in Overlapping Areas Between Predators and Prey and Identification of Conservation Gaps

**FIGURE 5:**
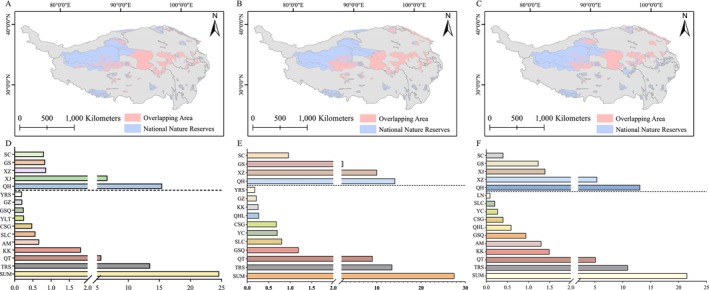
Distribution of Tibetan fox (A), red fox (B) and plateau pika (C) in the QTP national reserve. The bar charts below the dashed lines (D–F) represent the proportions of Tibetan foxes, red foxes, and plateau pikas in different national protected areas on the QTP, relative to the total across all protected areas (showing only the top ten areas with the highest proportions). The bar charts above the dotted line represent the proportions of Tibetan fox, red fox and plateau pika in the total protected areas of Qinghai (QH), Xinjiang (XJ), Xizang (XZ), Gansu (GS) and Sichuan (SC), respectively. SUM represents the sum of the proportion of the three species in all protected areas, and the full names of other protected areas are Three‐River Source (TRS), Qiangtang (QT), Kekexili (KK), Altun Mountains (AM), Selin Co Lake (SLC), Changsha Gongma (CSG), Tibetan Middle Reaches of Yarlung Tsangpo River Valley (YLT), Gansu Qilian Mountains (GSQ), Gahai‐Zecha (GZ), Yanchiwan (YC), Yellow River Source Area (YRS), Qinghai Lake (QHL), Lop Nur (LN).

Our analysis indicated that the overlap area between the two predator–prey pairs increased under the climate scenarios of RCP2.6–2050s, RCP6.0–2070s, and RCP8.5–2050s, while decreasing under other scenarios. Furthermore, the spatial overlap between the two predator species decreased under the scenarios of RCP2.6–2070s, RCP4.5–2070s, and RCP8.5–2070s but increased under all other scenarios (Figure 5, Table 2). Additionally, the results showed that, currently, the suitable habitat for the three species had the highest proportion in the Sanjiangyuan National Park and the Qiangtang Nature Reserve. Relatively high proportions were also found in other reserves, such as the Altun Mountains, Hoh Xil, and Gansu Qilian Mountains (Table S3). Overall, reserves in Qinghai Province and Tibet provided more extensive suitable habitats for the species.

**TABLE 2:** Changes in overlap area between predators and prey under climate change.

Proportion(%)	RCP2.6–2050s	RCP2.6–2070s	RCP4.5–2050s	RCP4.5–2070s	RCP6.0–2050s	RCP6.0–2070s	RCP8.5–2050s	RCP8.5–2070s
Red fox and Plateau pika	0. 83	−0. 93	−0. 51	−1.11	−0.64	0.06	0.25	−1.29
Tibetan fox and Plateau pika	0. 54	−0. 88	−0. 03	−1.00	−0.23	0.63	0.88	−0.52
Red fox and Tibetan fox	1.73	−0.45	0.54	−0.33	0.62	0.65	0.96	−1.58

## 4 Discussion

The QTP, a hotspot for biodiversity, demands a deep understanding of species distribution and food chain integrity to maintain its ecosystem stability. Due to the vastness and inaccessibility of certain areas on the plateau, direct species surveys are inherently limited. Thus, employing ecological niche distribution models like MaxEnt is highly beneficial for analyzing habitat suitability and exploring species' adaptive mechanisms to environmental factors, providing essential insights for conservation efforts and ecosystem management (Li et al. 2023; Zhang et al. 2021). We used Maxent as the model for predicting the suitability distribution of red fox, Tibetan fox and plateau pika, and the accuracy of the model prediction was greatly improved through the parameter optimization of the model. In this study, all environmental variables included in the model analysis were climate‐related factors, reflecting their widely acknowledged role in determining species distributions (He et al. 2019). These variables interact intricately to influence species' ecological niches and habitat preferences, ultimately shaping their geographic ranges (Cetin et al. 2023). Temperature emerged as a more significant determinant than precipitation, likely due to its direct effects on animal growth and reproduction (Zhang et al. 2019). Furthermore, temperature and precipitation indirectly influenced vegetation phenology and growth by affecting photosynthesis and soil moisture (Shi et al. 2023), which in turn impacted the survival of the herbivorous pika and the distribution of its predators, the Tibetan and red foxes. Significant differences in climate tolerance among the species were observed in mean diurnal temperature range (bio2), annual precipitation (bio12), and precipitation seasonality (bio15). Plateau pika demonstrated a broader tolerance for mean diurnal temperature range and precipitation variability and was more adapted to arid conditions than the foxes. Notably, excessive rainfall adversely affected pika survival (Pan 2016), emphasizing this species unique adaptations compared to the other two species.

The Tibetan fox exhibits a highly specialized dependence on plateau pikas, making it more reliant on pika population dynamics and habitat changes. In contrast, the red fox, as a generalist predator, has a more diverse diet. The future climate change projected in this study will intensify the pressure on the survival space of both the red fox and Tibetan fox over time. The range centroids of both species shifted toward higher latitudes, with future range reductions primarily concentrated in the western and southern regions. Furthermore, altitude‐based migration trends indicated a shift toward higher elevations. This pattern aligns with the migration direction of many species under climate change (Chen et al. 2011; Freeman and Class Freeman 2014), suggesting that higher latitudes and altitudes will provide refuges for these species. For the plateau pika, its future habitat area showed uncertainty under different scenarios. In the scenario of moderate greenhouse gas emissions, its suitable habitat exhibited a slight decreasing trend, with a general shift toward lower elevations. This outcome was likely linked to the uncertainties in plant phenology and precipitation changes brought about by climate change (Shen et al. 2015), which could further affect the availability of food resources for the pikas.

Based on the research findings, we observed that the changes in suitable habitats for the two fox species under climate change scenarios did not align closely with those of the plateau pika. This raises important questions about whether such discrepancies might lead to a reduction in habitat overlap between predators and their prey. Furthermore, it prompts further investigation into whether this mismatch could negatively impact the availability of food resources for predators, thereby exacerbating the ecological pressures they already face due to climate‐induced habitat shifts. Plateau pika serves as a primary prey not only for these foxes but also for other predators like lynxes, snow leopards, and birds of prey (Smith et al. 2019; Zhang et al. 2019). Although plateau pikas are considered “ecosystem engineers” of the QTP, predation plays a crucial role in regulating their population density, preventing overgrazing, and maintaining the stability of the grassland ecosystem (Smith and Foggin 1999). In this study, we analyzed and compared habitat overlap between predators and prey under future scenarios relative to current conditions. The results showed no consistent patterns in changes to overlap areas. Small fluctuations were observed across different years and climate scenarios. However, the proportion of reduced overlap between red foxes and plateau pikas was generally higher than that between Tibetan foxes and plateau pikas. Compared to the Tibetan fox, which relies more heavily on plateau pikas, the red fox is less affected by climate change. An important ecological concern is that competition from red foxes may impact the survival of Tibetan foxes. By comparison, Tibetan foxes are more vulnerable to future climate change due to the more severe reduction in their suitable habitats. Additionally, under certain climate scenarios and timeframes, such as RCP4.5–2050s and RCP6.0–2050s, where the suitable distribution areas of both predator–prey pairs decrease while the overlap between the two predator species increases, competition from red foxes poses an even greater threat to Tibetan foxes. From a conservation perspective, Qinghai's Sanjiangyuan National Park and Xizang's Changtang Nature Reserve have demonstrated significant advantages in providing habitat security. As such, these areas are identified as priority regions for the protection of all three species, underscoring their critical role in preserving this ecosystem.

This study provides valuable insights into the spatial dynamics of Tibetan foxes, red foxes, and plateau pikas on the Qinghai‐Tibetan Plateau under future climate change scenarios. It highlights how the synchronized changes between predators and prey underscore the potential threats posed by climate change to high‐altitude ecosystems. To address these threats, the study emphasizes the importance of protecting critical habitats and potential climate refugia identified in the analysis. As the plateau is a climate‐sensitive region, its species distributions are particularly vulnerable to climatic and environmental factors (Hu et al. 2024; Yin et al. 2022). The climate change research framework used in this study for predator–prey relationships is applicable to studying other predator–prey interactions and the overall food chain structure within the Qinghai‐Tibetan Plateau ecosystem. However, this study has certain limitations. First, our research primarily relies on climate variables and does not account for other environmental factors such as land‐use changes and human disturbances, which may significantly influence species distributions. Second, the accuracy of the MaxEnt model depends on the quality and availability of species distribution data. Future studies should incorporate more extensive field survey data to enhance the reliability of predictions. Therefore, future research should integrate broader environmental data, including land‐use changes and human disturbances (Shi et al. 2023; Zhang et al. 2015), to comprehensively assess the potential shifts in key species distributions under future scenarios.

## 5 Conclusion

Climate change has the potential to significantly impact the suitable distribution areas of species, reducing their current habitats while introducing new suitable regions. This study evaluated the range shifts of suitable habitats for red foxes, Tibetan foxes, and plateau pikas on the QTP, examining habitat loss and potential expansion under future climate conditions. It also analyzed the spatial and altitudinal migration patterns and distances of these species and assessed predator–prey dynamics under changing climates. Furthermore, the study investigated the extent of species' habitats within national parks on the plateau to evaluate conservation coverage and identify priority protection areas. These findings provide crucial insights into the spatial dynamics of QTP species under future climate change scenarios. They underscore the potential threats to high‐altitude ecosystems and offer evidence‐based recommendations for developing effective biodiversity conservation strategies.

## Author Contributions

**Jingjie Zhang:** conceptualization (equal), funding acquisition (equal), methodology (equal), writing – original draft (lead), writing – review and editing (lead). **Feng Jiang:** conceptualization (equal), investigation (equal), software (equal), writing – review and editing (equal). **Hongmei Gao:** conceptualization (equal), data curation (equal), investigation (equal). **Haifeng Gu:** investigation (equal), visualization (equal). **Pengfei Song:** data curation (equal), investigation (equal). **Tongzuo Zhang:** conceptualization (equal), funding acquisition (equal), project administration (equal), resources (lead), supervision (lead).

## Ethics Statement

This research does not involve any human participants or animals.

## Conflicts of Interest

The authors declare no conflicts of interest.

## Supporting information


**Data S1.** Supporting Information.

## Data Availability

The 19 bioclimatic variables used in this study were sourced from the WorldClim Global Climate Data platform (http://www.worldclim.org/). Species occurrence data for the Tibetan fox, red fox, and Plateau pika were partially obtained from the Global Biodiversity Information Facility (GBIF) database (https://www.gbif.org) and the Species Diversity Data Platform (SDDP) database (http://www.especies.cn/). Data sharing is not applicable to this study, as no new data were generated or analyzed during this study.
