# Supplementary material for: Dynamics of Suitable Habitats for Typical Predators and Prey on the Qinghai‐Tibet Plateau Driven by Climate Change: A Case Study of Tibetan Fox, Red Fox, and Plateau Pika
Source: Ecol Evol. 2025 Apr 18;15(4):e71295. doi: 10.1002/ece3.71295 (PMC12008021; doi:10.1002/ece3.71295)
Supplement: Supplementary file 1 — Data S1. Supporting Information. [file ECE3-15-e71295-s001.docx]

**Habitat dynamics of Tibetan foxes, red foxes and plateau pikas in the Qinghai-Tibet Plateau driven by climate change**

**Table S1 The environment variable**

| Code | Description | Unit |
| --- | --- | --- |
| bio1 | Annual mean temperature | ℃ |
| bio2 | Mean diurnal range (Mean of monthly (max.temp.-min.temp.) | ℃ |
| bio3 | Isothermality (bio2 / bio7) (× 100) |  |
| bio4 | Temperature seasonality (standard deviation*100) |  |
| bio5 | Max temperature of the warmest month | ℃ |
| bio6 | Min temperature of the coldest month | ℃ |
| bio7 | Temperature annual range (bio5- bio6) | ℃ |
| bio8 | Mean temperature of the wettest quarter | ℃ |
| bio9 | Mean temperature of the driest quarter | ℃ |
| bio10 | Mean temperature of the warmest quarter | ℃ |
| bio11 | Mean temperature of the coldest quarter | ℃ |
| bio12 | Annual precipitation | mm |
| bio13 | Precipitation of the wettest month | mm |
| bio14 | Precipitation of the driest month | mm |
| bio15 | Precipitation seasonality (Coefficient of variation) |  |
| bio16 | Precipitation of the wettest quarter | mm |
| bio17 | Precipitation of the driest quarter | mm |
| bio18 | Precipitation of the warmest quarter | mm |
| bio19 | Precipitation of coldest quarter | mm |

**Table S2 Percent contribution and permutation importance of each environmental variable of three species**

| Species | Code | Percent contribution |
| --- | --- | --- |
| Red fox | bio1 | 11.68 ± 1.04 |
|  | bio2 | 26.25 ± 1.04 |
|  | bio3 | 11.04 ± 1.42 |
|  | bio4 | 22.63 ± 0.76 |
|  | bio12 | 10.96 ± 1.26 |
|  | bio14 | 1.11 ± 0.52 |
|  | bio15 | 16.33 ± 1.24 |
| Tibetan fox | bio1 | 21.97 ± 1.26 |
|  | bio2 | 6.27 ± 1.36 |
|  | bio3 | 1.16 ± 0.27 |
|  | bio7 | 37.71 ± 1.80 |
|  | bio12 | 19.74 ± 0.80 |
|  | bio14 | 7.82 ± 1.52 |
|  | bio15 | 5.32 ± 0.51 |
| Plateau pika | bio1 | 21.76 ± 0.65 |
|  | bio2 | 23.61 ± 2.03 |
|  | bio3 | 12.00 ± 1.05 |
|  | bio4 | 10.66 ± 0.74 |
|  | bio12 | 14.96 ± 1.40 |
|  | bio14 | 2.10 ± 0.57 |
|  | bio15 | 11.74 ± 0.81 |
|  | bio19 | 3.15 ± 0.70 |

**Table S3 The proportion of Tibetan foxes, red foxes, and plateau pikas in different national nature reserves relative to the total area of all national nature reserves on the QTP (only listing the top 10 reserves with the highest proportions) and the total proportion of the three species**

| National protected areas on the QTP | Red fox | Tibetan fox | Plateau pika |
| --- | --- | --- | --- |
| Three-River Source | 13.52 | 13.47 | 10.91 |
| Qiangtang | 5.67 | 9.03 | 5.15 |
| Kekexili | 1.82 | 0.26 | 1.50 |
| Gansu Qilian Mountains | — | 1.20 | 0.94 |
| Yanchiwan | — | 0.70 | 0.27 |
| Altun Mountains | 0.67 | — | 1.30 |
| Selin Co Lake | 0.57 | 0.81 | 0.21 |
| Qinghai Lake | — | 0.28 | 0.59 |
| Changsha Gongma | 0.48 | 0.68 | 0.40 |
| Tibetan Middle Reaches of Yarlung Tsangpo River Valley | 0.26 | — | — |
| Gansu Qilian Mountains | 0.25 | — | — |
| Gahai-Zecha | 0.21 | 0.22 | — |
| Yellow River Source Area | 0.20 | 0.18 | — |
| Lop Nur | — | — | 0.09 |
| Sum | 24.64 | 21.53 | 21.53 |


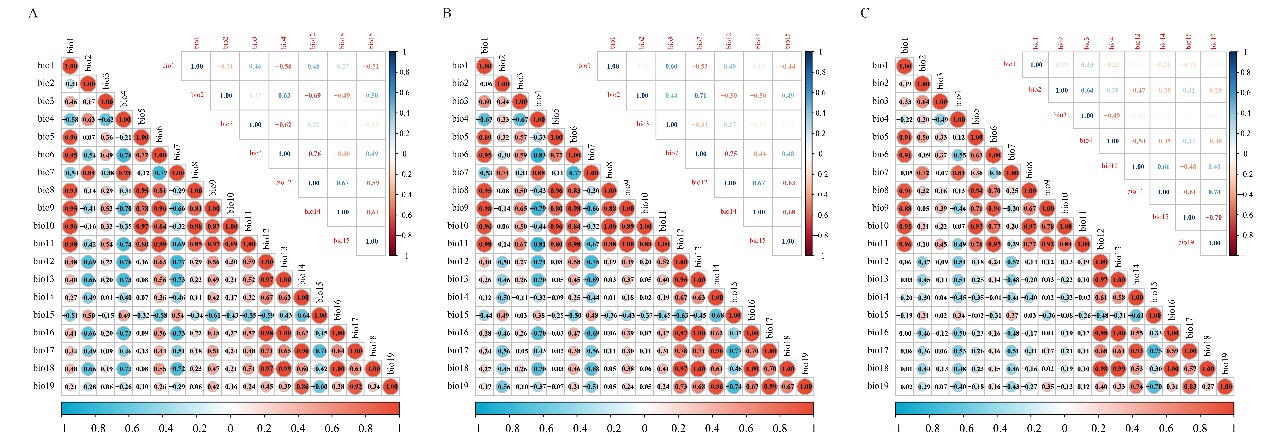


**Fig. S1. The environmental variables of the red fox (A), Tibetan fox (B) and plateau pika (C) included in the model were screened by variable correlation**


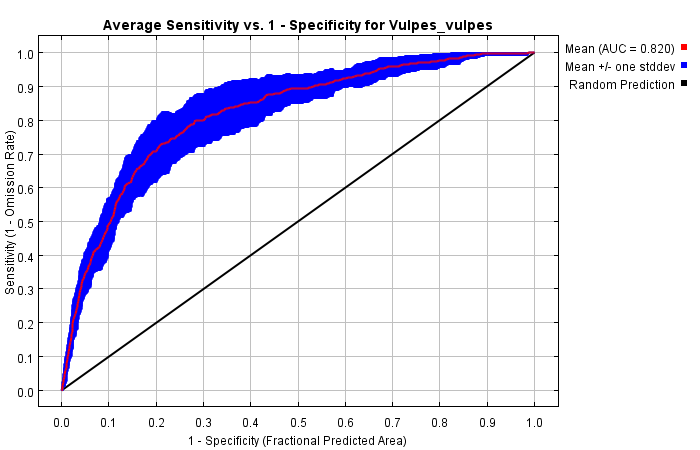


**Fig. S2. The receiver operating characteristic (ROC) curve for *Vulpes vulpes***


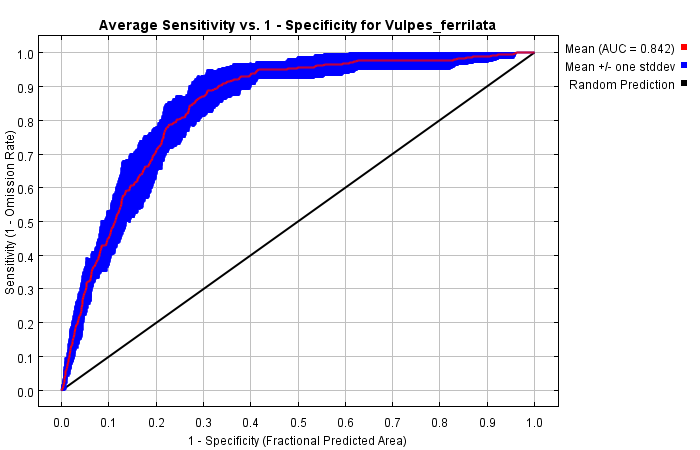


**Fig. S3. The receiver operating characteristic (ROC) curve for *Vulpes ferrilata***


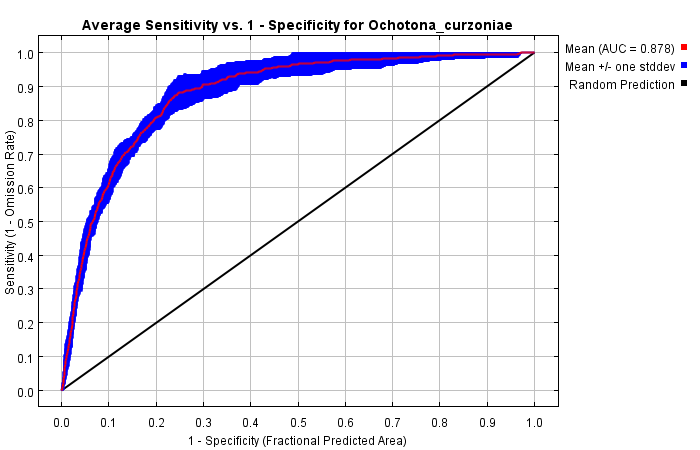


**Fig. S4. The receiver operating characteristic (ROC) curve for *Ochotona curzoniae***
